# A Phase I trial of talazoparib in patients with advanced hematologic malignancies

**DOI:** 10.2217/ijh-2021-0004

**Published:** 2021-10-22

**Authors:** Ajay K Gopal, Rakesh Popat, Ryan J Mattison, Tobias Menne, Adrian Bloor, Terry Gaymes, Asim Khwaja, Mark Juckett, Ying Chen, Matthew J Cotter, Ghulam J Mufti

**Affiliations:** 1 Division of Medical Oncology, Department of Medicine, University of Washington, Seattle, Clinical Research Division, Fred Hutchinson Cancer Research Center, Seattle, WA 98109, USA; 2 National Institute for Health Research University College London Hospitals Clinical Research Facility, University College London Hospitals NHS Foundation Trust, London, UK; 3 Carbone Cancer Center, University of Wisconsin, Madison, WI, USA; 4 Department of Hematology, Freeman Hospital, Newcastle Upon Tyne Hospitals NHS Foundation Trust, Newcastle Upon Tyne, UK; 5 The Christie NHS Foundation Trust, University of Manchester, Manchester, UK; 6 Department of Biomolecular Science, Kingston University, London, UK; 7 University College London Cancer Institute & University College London Hospitals NHS Foundation Trust, London, UK; 8 Pfizer Inc., New York, NY, USA; 9 Department of Hematology, King's College London, King's College Hospital NHS Foundation Trust, London, UK

**Keywords:** *BRCA1/2* mutations, DNA damage response, hematologic malignancy, poly(ADP-ribose) polymerase inhibition, talazoparib

## Abstract

**Aim:**

The objective of this study was to establish the maximum tolerated dose (MTD), safety, pharmacokinetics, and anti-leukemic activity of talazoparib.

**Patients & methods:**

This Phase I, two-cohort, dose-escalation trial evaluated talazoparib monotherapy in advanced hematologic malignancies (cohort 1: acute myeloid leukemia/myelodysplastic syndrome; cohort 2: chronic lymphocytic leukemia/mantle cell lymphoma).

**Results:**

Thirty-three (cohort 1: n = 25; cohort 2: n = 8) patients received talazoparib (0.1–2.0 mg once daily). The MTD was exceeded at 2.0 mg/day in cohort 1 and at 0.9 mg/day in cohort 2. Grade ≥3 adverse events were primarily hematologic. Eighteen (54.5%) patients reported stable disease.

**Conclusion:**

Talazoparib is relatively well tolerated in hematologic malignancies, with a similar MTD as in solid tumors, and shows preliminary anti leukemic activity.

Clinical trial registration: NCT01399840 (ClinicalTrials.gov)

## Lay abstract

The objective of this study was to define the highest dose of talazoparib that people with various types of leukemia (mainly various blood cancers) could tolerate. People were assigned into two cohorts based on their type of leukemia: cohort 1 included 25 people with acute myeloid leukemia or myelodysplastic syndrome; cohort 2 included 8 people with chronic lymphocytic leukemia or mantle cell lymphoma. Similar to what researchers observed for people with solid tumors, the highest tolerated dose was 1.35 mg per day in cohort 1, and it was estimated to be ∼0.9 mg per day in cohort 2. Side effects that occurred during the study were expected, given the types of leukemia being treated. Talazoparib also showed promising anti leukemic effects in some patients.

## Tweetable abstract

In this Phase I talazoparib trial in hematologic malignancies (cohort 1: AML/MDS, n = 25; cohort 2: CLL/MCL, n = 8), the maximum tolerated dose was exceeded at 2.0 and 0.9 mg/day in cohorts 1 and 2, respectively. Stable disease and transfusion independence were also observed.

## Introduction

Hematologic malignancies are heterogeneous blood cancers that vary in incidence, etiology, and survival [1]. Because of population growth and increased longevity, there has been an increase in the global incidence of hematologic malignancies in recent years [2]. Indeed, between 2006 and 2016, the global incidence of leukemia and non-Hodgkin lymphoma increased by 26% (370,482 to 466,802 cases) and 45% (319,078 to 461,164 cases), respectively [2].

Similar to most other cancers, hematologic malignancies may exhibit deficiencies in DNA damage response (DDR) pathways, including homologous recombination, that render cells highly dependent on alternative DNA repair mechanisms such as those mediated by PARP enzymes 1 and 2 [3,4]. There is substantial clinical evidence demonstrating that PARP inhibitors can effectively treat solid tumors with DDR deficiencies, whereby tumor cells with *BRCA1/2* mutations are selectively killed via synthetic lethality [5,6]. Although clinical evidence for PARP inhibition in hematologic malignancies is limited, there are considerable pre-clinical data pointing to an association between hematologic cancers and several DDR gene mutations. For example, alterations in *ATM* have been implicated in mantle cell lymphoma (MCL), chronic lymphocytic leukemia (CLL), and myelodysplastic syndrome (MDS) [4,7]. Additionally, mutations in *CHEK2* and *BRCA2* have been linked to CLL and MDS [4,8,9], *RAD51* in acute myeloid leukemia (AML) and MDS [10,11], *MLH1* in MDS [12,13], and *BRCA1* in AML [4]. Moreover, both *in vitro* and *in vivo* studies have demonstrated that ATM-deficient lymphoid tumor cells, i.e., CLL and MCL, are sensitive to PARP inhibition, with data showing cytotoxicity as well as reduced tumor growth and prolonged survival in animal models [14–17]. In addition, low *BRCA1* mRNA expression has been associated with sensitivity to PARP inhibition, both in CLL and AML [18,19].

The PARP inhibitor talazoparib has demonstrated efficacy as a monotherapy in cancers with germline *BRCA1/2* mutations [20–22], and it has also shown higher PARP-trapping activity *in vitro* compared with other PARP inhibitors [3,23,24]. A Phase I, dose-escalation trial of talazoparib in patients with germline *BRCA1/2* mutations and solid tumors (NCT01286987) established the maximum tolerated dose (MTD) as 1 mg once daily [22]. In the Phase III EMBRACA trial (NCT01945775) that followed, patients with germline *BRCA1/2*-mutated HER2- advanced breast cancer treated with 1 mg once daily talazoparib had significantly longer progression-free survival and clinically meaningful improvements in patient-reported outcomes compared with physician's choice of chemotherapy [20], leading to regulatory approvals for this indication in the US, EU, and multiple other countries [25,26]. In addition, talazoparib is in clinical development for metastatic castration-resistant prostate cancer (mCRPC), including as monotherapy in previously treated patients with DDR-deficient mCRPC (NCT03148795) [27], and as a first-line treatment in combination with enzalutamide in molecularly unselected patients with mCRPC (NCT03395197) [28].

This Phase I, two-cohort, dose-escalation trial of talazoparib monotherapy in molecularly unselected patients with advanced hematologic malignancies (ClinicalTrials.gov Identifier: NCT01399840) was conducted prior to the approval of talazoparib to treat *BRCA1/2*-mutated HER2- advanced breast cancer [25,26] and in parallel with the solid tumor Phase I study (NCT01286987) that determined the talazoparib MTD at 1 mg once daily [22]. Given the pre-clinical rationale for the use of PARP inhibitors in hematologic malignancies, it was of interest to evaluate the MTD, safety, and anti leukemic effects of talazoparib in this hematology-specific Phase I study. The primary objective of this study was to establish the MTD of talazoparib in patients with AML, MDS, CLL, or MCL, with secondary objectives including the evaluation of the safety, pharmacokinetics (PK), and preliminary anti-leukemic activity of talazoparib in these disorders.

## Patients & methods

### Study population

The study was divided into two cohorts as it was anticipated that impaired hematopoiesis and, therefore, the toxicity profile and tolerability, may vary between different hematologic malignancies: cohort 1 of the study enrolled patients with AML (relapsed, refractory, or newly diagnosed by WHO's classification [>20% myeloblasts]) or patients with MDS who had failed or declined standard-of-care therapy, while cohort 2 included patients with CLL or MCL, who had relapsed, were refractory or intolerant of standard treatment, or had declined standard therapy.

Patients enrolled in the study were ≥18 years of age, with an Eastern Cooperative Oncology Group (ECOG) performance status ≤1. Other inclusion criteria included adequate organ function, defined as serum AST and ALT values ≤2.5 × upper limit of normal (ULN) and total serum bilirubin ≤1.5 × ULN (≤3 × ULN for Gilbert's syndrome). Patients also needed to demonstrate recovery from acute toxicity due to prior treatment. Key exclusion criteria included acute promyelocytic leukemia and certain disease-specific criteria; these included marrow cellularity <25% or circulating blasts >50 × 10^9^/L for AML, and platelet count <50 × 10^9^/L and neutrophil count <1 × 10^9^/L for MCL/CLL (unless decreased counts were secondary to bone marrow effacement due to leukemia or lymphoma, splenomegaly, or autoimmune thrombocytopenia). Additional key exclusion criteria included prior allogeneic or autologous bone marrow transplant <6 months before cycle 1 day 1 and/or the presence of graft-versus-host disease (acute or chronic; allogeneic bone marrow transplant only). Exclusionary prior treatment included anti-leukemia treatment within 14 days or hydroxyurea treatment within 7 days of first dose for AML; anti-lymphoma/leukemia treatment within 28 days of first dose for CLL, MCL, or MDS patients; and transfusion/hematopoietic growth factors within 7 days of first dose for CLL and MCL patients.

### Study design

This study was an open-label, two-cohort, Phase I, dose-escalation study of once-daily talazoparib monotherapy in patients with advanced hematologic malignancies (ClinicalTrials.gov Identifier: NCT01399840) conducted at seven sites in the US and the UK between July 2011 and November 2013. Enrollment and dose escalation were conducted independently in each cohort of the study. Talazoparib was administered orally, once a day for repeated 21-day cycles under fasting conditions. Each cohort consisted of two stages: dose escalation (stage 1) and dose expansion (stage 2). Dose escalation followed a standard 3 + 3 dose-escalation design [29], where the first patient in each group was observed from cycle 1 day 1 through cycle 1 day 8 before two to five additional patients were treated in that dose level. Groups of three to six patients were enrolled in each cohort to receive increasing talazoparib doses; each dose level had to be expanded to six patients if a dose-limiting toxicity (DLT) was observed in one patient. Planned talazoparib dose levels included 0.1, 0.2, 0.3, 0.45, 0.9, 1.35, and 2.0 mg/day.

### Study endpoints

The primary objective of the study was to establish the MTD of daily oral talazoparib in molecularly unselected patients with AML or MDS (cohort 1) and in patients with CLL or MCL (cohort 2). The MTD was defined as the highest dose level at which no more than one of six patients experienced a DLT. A DLT was defined as any of the following treatment-related toxicities occurring during cycle 1: in cohort 1, hematologic adverse events (AEs) included bone marrow hypoplasia (≤10% cellularity) lasting ≥28 days. Non-hematologic toxicities included any Grade ≥3 AE except for the following: (1) a non-hematologic Grade 3 laboratory AE that is asymptomatic and rapidly reversible (returned to baseline or to Grade ≤1 within 3 days) unless identified as clinically relevant by the investigator; (2) Grade 3 nausea, vomiting, and/or diarrhea responsive to medications within 24 h; (3) Grade 3 tumor lysis syndrome; (4) Grade 3 stomatitis that resolves within 7 days; or (5) Grade 3 fatigue unless ≥2 grade increase from baseline lasting >3 days. Missing ≥4 doses of talazoparib during cycle 1 for treatment-related non-hematologic toxicity was also defined as a DLT. In cohort 2, hematologic AEs included Grade 4 thrombocytopenia persisting for >7 days and/or resulting in Grade ≥2 hemorrhage, or Grade 4 neutropenia lasting >7 days and/or associated with fever (hematologic AEs for patients with bone marrow effacement [i.e., platelet count ≤50 × 10^9^/L and neutrophil count <1 × 10^9^/L] included >50% decrease in platelet count persisting for >7 days or resulting in Grade ≥2 hemorrhage, or >50% decrease in neutrophil count and/or associated with fever or systemic infection). For non-hematologic AEs, the same criteria used for cohort 1 were used to define DLT.

AEs were coded in accordance with The Medical Dictionary for Regulatory Activities, version 16.1. Treatment-emergent AEs (TEAEs) were defined as AEs that were new, increased in frequency, or worsened in severity, after the first talazoparib dose. AEs counted as drug-related had been classified by investigators as possibly or probably related to talazoparib.

The secondary objectives of the study were to assess the safety, PK, and preliminary anti-leukemic activity of talazoparib in cohorts 1 and 2. Objective response, duration of objective response, and time to progression or treatment failure were recorded. Response was measured by the following response criteria: the European leukemiaNet for subjects with AML [30], the International Working Group for Prognosis in MDS for subjects with MDS [31,32], the International Workshop on Chronic Lymphocytic leukemia (iwCLL) [33] for subjects with CLL, and the Revised Response Criteria for MCL [34].

### Dose modifications

Patients experiencing a DLT during cycle 1 could resume talazoparib treatment at the same or reduced dose after recovery to baseline or Grade ≤1 within 21 days of treatment interruption. Patients not experiencing a DLT during cycle 1 could receive additional treatment until disease progression, treatment failure, or relapse. Patients with persisting non-hematologic Grade 1/2 toxicity were dose reduced ∼25%. Dosing was stopped for patients with a Grade 3/4 non-hematologic toxicity and resumed with a 25%–50% dose reduction if toxicity resolved to Grade 0/1 after >7 days, or returned to baseline. Patients with non-hematologic Grade 3 toxicity that resolved to Grade 0/1 in <7 days could resume with ∼25% reduction.

### PK sample collection & assessments

Plasma and urine samples were assayed for talazoparib concentrations by Covance Laboratories Inc., using validated high performance liquid chromatography with tandem mass spectrometry detection methods [22]. The lower limits of quantitation for serum and urine were 5.0 pg/ml and 25.0 pg/ml, respectively. Evaluated PK parameters included maximum concentration (C_max_), minimum concentration (C_min_), time to maximum plasma concentration (T_max_), area under the curve from time 0 to last measurable concentration (AUC_0–t_), area under the curve from time 0 to 24 h post-dose (AUC_0–24_), amount of drug excreted into the urine from time 0 to 24 h (Ae_0–24_), fraction of administered drug excreted in the urine from time 0 to 24 h (Fe_0–24_), and average renal clearance from time 0 to 24 h (ARC_0–24_); ARC_0–24_ was calculated as (Ae_0–24_/AUC_0–24_). PK parameters were calculated using Phoenix^™^ WinNonlin^®^ 6.1.

### Statistical analyses

All analyses in this open-label, dose-finding study were descriptive in nature. Descriptive summaries of continuous variables, including the mean, standard deviation (SD), median, range, and, where appropriate, 95% CI and/or interquartile range, were determined using the Statistical Analysis Software (SAS), version 9.2. Descriptive summaries of categorical variables included the number of patients and percentages. Data were summarized with respect to the dose level at which the patients started treatment. Cohorts were defined as all patients who began treatment at a given dose level.

For determination of the MTD, only the treated evaluable patients were included. No formal sample size calculations based on statistical power were performed. Descriptive statistics were used to characterize all outcomes.

Summary statistics for plasma and urine talazoparib concentrations and PK parameters were calculated by grouping subjects in cohorts 1 and 2 together to provide a larger sample size at each dose level.

## Results

### Patients

**Figure 1.**
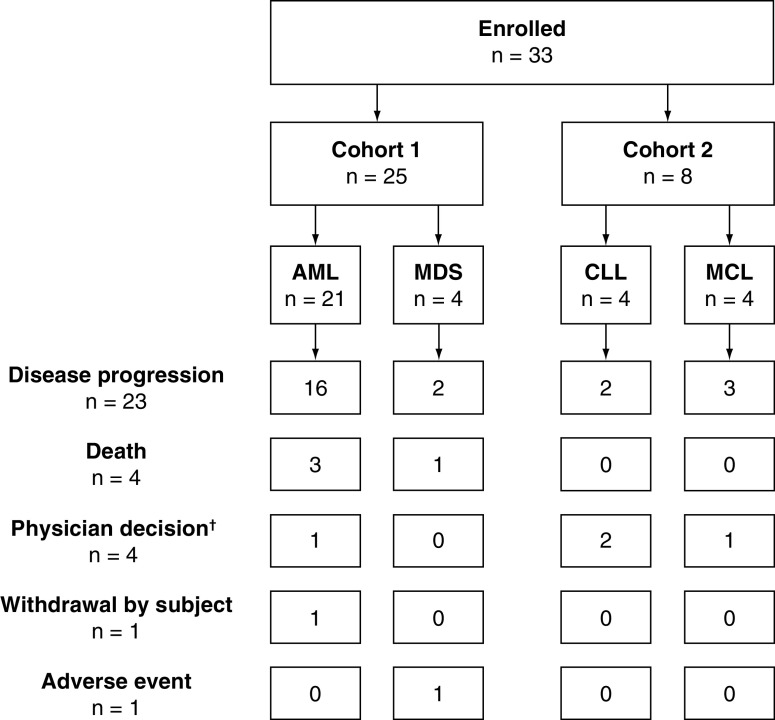
Subject disposition. ^†^Of the patients withdrawn from the study due to physician decision, two patients (6.1%) were withdrawn due to an apparent lack of therapeutic response, one patient (3.0%) was withdrawn because they did not tolerate therapy, and one patient (3.0) was withdrawn to begin hydroxyurea treatment. AML: Acute myeloid leukemia; CLL: Chronic lymphocytic leukemia; MCL: Mantle cell lymphoma; MDS: Myelodysplastic syndrome.

A total of 33 patients (23 males, ten females) were treated with talazoparib during the study and included in the safety analyses. There were 25 patients in cohort 1 (21 with AML, four with MDS) and eight patients in cohort 2 (four with CLL, four with MCL; Figure 1, Supplementary Table 1).

The median age of patients was 70.0 and 75.5 years in cohort 1 and cohort 2 of the study, respectively (Table 1). Patients were predominantly males and white (Supplementary Table 1). The median number of previous chemotherapy regimens was three (maximum = seven) and six (maximum = 13) in cohorts 1 and 2, respectively.

**Table 1.** Participant demographics and baseline clinical characteristics (safety population).

Dose level, mg/day	Cohort 1								Cohort 2		
	0.10 (n = 3)	0.20 (n = 3)	0.30 (n = 5)	0.45 (n = 3)	0.90 (n = 4)	1.35 (n = 3)	2.00 (n = 4)	All (n = 25)	0.10 (n = 3)	0.90 (n = 5)	All (n = 8)
Median age (range), years	56.0 (37, 69)	79.0 (72, 83)	71.0 (66, 76)	62.0 (33, 71)	67.5 (22, 74)	70.0 (40, 74)	68.5 (65, 71)	70.0 (22, 83)	82.0 (68, 86)	75.0 (49, 76)	75.5 (49, 86)
Sex, n (%) Female Male	1 (33.3) 2 (66.7)	1 (33.3) 2 (66.7)	1 (20.0) 4 (80.0)	1 (33.3) 2 (66.7)	2 (50.0) 2 (50.0)	1 (33.3) 2 (66.7)	1 (25.0) 3 (75.0)	8 (32.0) 17 (68.0)	0 (0.0) 3 (100)	2 (40.0) 3 (60.0)	2 (25.0) 6 (75.0)
Race, n (%)^†^ White Asian Black Anglo–Indian Turkish	2 (66.7) 0 (0.0) 0 (0.0) 1 (33.3) 0 (0.0)	3 (100) 0 (0.0) 0 (0.0) 0 (0.0) 0 (0.0)	4 (80.0) 0 (0.0) 0 (0.0) 0 (0.0) 1 (20.0)	3 (100) 0 (0.0) 0 (0.0) 0 (0.0) 0 (0.0)	4 (100) 0 (0.0) 0 (0.0) 0 (0.0) 0 (0.0)	3 (100) 0 (0.0) 0 (0.0) 0 (0.0) 0 (0.0)	4 (100) 0 (0.0) 0 (0.0) 0 (0.0) 0 (0.0)	23 (92.0) 0 (0.0) 0 (0.0) 1 (4.0) 1 (4.0)	3 (100) 0 (0.0) 0 (0.0) 0 (0.0) 0 (0.0)	4 (80.0) 1 (20.0) 0 (0.0) 0 (0.0) 0 (0.0)	7 (87.5) 1 (12.5) 0 (0.0) 0 (0.0) 0 (0.0)
BMI, kg/m^2^ n Median (range)	3 24.9 (20.6, 27.9)	2 21.1 (19.8, 22.4)	3 28.4 (24.8, 35.9)	3 30.8 (28.8, 47.2)	4 24.7 (21.1, 33.3)	3 25.8 (24.1, 29.4)	4 27.8 (25.0, 29.5)	22 27.3 (19.8, 47.2)	3 27.2 (19.7, 29.0)	5 23.3 (18.3, 32.9)	8 24.4 (18.3, 32.9)
ECOG, n (%)^‡^ 0 1	1 (33.3) 2 (66.7)	0 (0.0) 3 (100)	2 (40.0) 3 (60.0)	0 (0.0) 3 (100)	0 (0.0) 4 (100)	1 (33.3) 2 (66.7)	1 (25.0) 3 (75.0)	5 (20.0) 20 (80.0)	1 (33.3) 2 (66.7)	4 (80.0) 1 (20.0)	5 (62.5) 3 (37.5)

† None of the patients were of Hispanic ethnicity.

‡ ECOG performance status 0 = fully active, able to carry on all pre-disease performance without restriction; ECOG performance status 1 = restricted in physically strenuous activity but ambulatory and able to carry out work of a light or sedentary nature.

ECOG: Eastern Cooperative Oncology Group.

All patients completed the study as required by the protocol. Following study completion, primary reasons for ending treatment included disease progression (69.7% [n = 23]), withdrawal by subject (3.0% [n = 1]), physician decision (12.1% [n = 4]), withdrawal due to an AE (3.0% [n = 1]) and death (12.1% [n = 4]; Figure 1).

### Exposure to talazoparib

The median talazoparib exposure duration for patients in cohort 1 and cohort 2 of the study was 49.0 days (range: 7.0–484.0) and 34.5 days (range: 13.0–202.0), respectively (Supplementary Table 2). The mean daily dose of talazoparib administered to patients in cohort 1 and cohort 2 of the study was 0.74 mg (SD: 0.61 mg) and 0.52 mg (SD: 0.39 mg), respectively (Supplementary Table 2).

### Maximum tolerated dose

Within cohort 1, all seven dose groups (0.1–2.0 mg/day) were explored, with four patients (three with AML, one with MDS) receiving the highest dose of 2.0 mg/day (Supplementary Table 1). DLTs in cycle 1 were observed in two patients (Table 2): at the 2.0 mg dose, one patient with MDS experienced febrile neutropenia and one patient with AML experienced neutropenic sepsis. The MTD was determined as 1.35 mg/day as no DLTs were observed in the three patients treated with this dose level.

**Table 2.** Dose-limiting toxicity adverse events (safety population).

Dose level, mg/day	Cohort 1								Cohort 2		
	0.10 (n = 3)	0.20 (n = 3)	0.30 (n = 5)	0.45 (n = 3)	0.90 (n = 4)	1.35 (n = 3)	2.00 (n = 4)	All (n = 25)	0.10 (n = 3)	0.90 (n = 5)	All (n = 8)
Any DLT AE, n (%)	0 (0.0)	0 (0.0)	0 (0.0)	0 (0.0)	0 (0.0)	0 (0.0)	2 (50.0)	2 (8.0)	0 (0.0)	2 (40.0)	2 (25.0)
Blood and lymphatic system disorders, n (%) Febrile neutropenia	0 (0.0)	0 (0.0)	0 (0.0)	0 (0.0)	0 (0.0)	0 (0.0)	1 (25.0)	1 (4.0)	0 (0.0)	0 (0.0)	0 (0.0)
Neutropenia	0 (0.0)	0 (0.0)	0 (0.0)	0 (0.0)	0 (0.0)	0 (0.0)	0 (0.0)	0 (0.0)	0 (0.0)	2 (40.0)	2 (25.0)
Infections and infestations, n (%) Neutropenic sepsis	0 (0.0)	0 (0.0)	0 (0.0)	0 (0.0)	0 (0.0)	0 (0.0)	1 (25.0)^†^	1 (4.0)	0 (0.0)	0 (0.0)	0 (0.0)

† One death due to neutropenic sepsis, which occurred in a patient with AML who received the highest dose of 2 mg talazoparib, was considered to be possibly drug-related by the investigator; further details on the patient's medical history can be found in the Supplementary Material.

AE: Adverse event; AML: Acute myeloid leukemia; DLT: Dose-limiting toxicity.

Within cohort 2, dose levels of 0.2, 0.3, and 0.45 mg/day were omitted because this dose range was well tolerated in the parallel dose-escalation study in patients with solid tumors [22]. As a result, only two dose groups (0.1 and 0.9 mg/day) were enrolled and treated, with five patients (three with CLL and two with MCL) receiving the maximum dose of 0.9 mg/day (Supplementary Table 1). DLTs in cycle 1 were observed in two patients (Table 2): at 0.9 mg/day, one patient with MCL and one patient with CLL experienced Grade 4 neutropenia. As no intermediate doses were investigated, the MTD was not further elucidated but was concluded to be exceeded at 0.9 mg/day in cohort 2 of the study.

### Adverse events

All patients experienced ≥1 TEAE (Table 3). When both cohorts were considered collectively, the most frequently reported TEAEs were fatigue, pyrexia, and extremity pain (Table 4). TEAEs considered by investigators to be possibly or probably related to talazoparib (drug-related) were reported for 23 (69.7%) patients (Table 3); among these, the most frequently reported TEAEs were fatigue, neutropenia, and nausea (Table 4).

**Table 3.** Summary of treatment-emergent adverse events with talazoparib (safety population).^†^

	Cohort 1								Cohort 2		
Dose level, mg/day	0.10 (n = 3)	0.20 (n = 3)	0.30 (n = 5)	0.45 (n = 3)	0.90 (n = 4)	1.35 (n = 3)	2.00 (n = 4)	All (n = 25)	0.10 (n = 3)	0.90 (n = 5)	All (n = 8)
Any AEs, n (%)	3 (100)	3 (100)	5 (100)	3 (100)	4 (100)	3 (100)	4 (100)	25 (100)	3 (100)	5 (100)	8 (100)
Any DLT AEs	0 (0.0)	0 (0.0)	0 (0.0)	0 (0.0)	0 (0.0)	0 (0.0)	2 (50.0)	2 (8.0)	0 (0.0)	2 (40.0)	2 (25.0)
Any study drug-related AEs^‡^	3 (100)	1 (33.3)	4 (80.0)	1 (33.3)	3 (75.0)	3 (100)	2 (50.0)	17 (68.0)	1 (33.0)	5 (100)	6 (75.0)
Any AEs of CTCAE Grade 3 or 4, n (%)	2 (66.7)	2 (66.7)	5 (100)	3 (100)	4 (100)	3 (100)	3 (75.0)	22 (88.0)	3 (100)	3 (60.0)	6 (75.0)
Any study drug-related AEs of CTCAE Grade of 3 or 4^‡^	1 (33.3)	0 (0.0)	3 (60.0)	0 (0.0)	2 (50.0)	2 (66.7)	2 (50.0)	10 (40.0)	1 (33.3)	3 (60.0)	4 (50.0)
Any AEs of CTCAE Grade of 5 (death), n (%)	0 (0.0)	2 (66.7)	1 (20.0)	2 (66.7)	3 (75.0)	1 (33.3)	3 (75.0)	12 (48.0)	1 (33.3)	0 (0.0)	1 (12.5)
Any study drug-related AEs of CTCAE Grade 5 (death)^‡^	0 (0.0)	0 (0.0)	0 (0.0)	0 (0.0)	0 (0.0)	0 (0.0)	1 (25.0)^§^	1 (4.0)^§^	0 (0.0)	0 (0.0)	0 (0.0)
Any SAEs, n (%)	2 (66.7)	2 (66.7)	4 (80.0)	3 (100)	4 (100)	2 (66.7)	4 (100)	21 (84.0)	2 (66.7)	2 (40.0)	4 (50.0)
Any study drug-related SAEs^‡^	0 (0.0)	0 (0.0)	0 (0.0)	0 (0.0)	1 (25.0)	0 (0.0)	2 (50.0)	3 (12.0)	0 (0.0)	0 (0.0)	0 (0.0)
Any SAEs of CTCAE Grade of 3 or 4	2 (66.7)	1 (33.3)	4 (80.0)	2 (66.7)	2 (50.0)	2 (66.7)	2 (50.0)	15 (60.0)	1 (33.3)	1 (20.0)	2 (25.0)
Any AEs leading to dose interruption or modification, n (%)	1 (33.3)	2 (66.7)	4 (80.0)	0 (0.0)	3 (75.0)	0 (0.0)	2 (50.0)	12 (48.0)	0 (0.0)	3 (60.0)	3 (37.5)
Any study drug-related AEs leading to dose interruption or modification^‡^	1 (33.3)	1 (33.3)	2 (40.0)	0 (0.0)	1 (25.0)	0 (0.0)	2 (50.0)	7 (28.0)	0 (0.0)	3 (60.0)	3 (37.5)
Any AEs leading to study drug withdrawn, n (%)	0 (0.0)	1 (33.3)	1 (20.0)	0 (0.0)	0 (0.0)	0 (0.0)	1 (25.0)	3 (12.0)	0 (0.0)	0 (0.0)	0 (0.0)
Any study drug-related AEs leading to study drug withdrawal^‡^	0 (0.0)	0 (0.0)	0 (0.0)	0 (0.0)	0 (0.0)	0 (0.0)	1 (25.0)	1 (4.0)	0 (0.0)	0 (0.0)	0 (0.0)

† Adverse events were coded with MedDRA version 16.1.

‡ AEs that were classified by the investigator as possibly or probably related to the study drug.

§ One death due to neutropenic sepsis, which occurred in a patient with AML who received the highest dose of 2 mg talazoparib, was considered to be possibly drug-related by the investigator; further details on the patient's medical history can be found in the Supplementary Material.

AE: Adverse event; AML: Acute myeloid leukemia; CTCAE: Common Terminology Criteria for Adverse Events; DLT: Dose-limiting toxicity; MedDRA: The Medical Dictionary for Regulatory Activities; SAE: Serious adverse event.

**Table 4.** Most frequently reported treatment-emergent adverse events with talazoparib (safety population).^†^

n = 33	Grade 1–2	Grade 3–4	All grades^‡^
Most frequently reported TEAEs,^§^ n (%)			
Fatigue	10 (30.3)	3 (9.1)	13 (39.4)
Pyrexia	12 (36.4)	0 (0.0)	12 (36.4)
Pain in extremity	10 (30.3)	1 (3.0)	11 (33.3)
Dyspnea	8 (24.2)	2 (6.1)	10 (30.3)
Epistaxis	9 (27.3)	1 (3.0)	10 (30.3)
Febrile neutropenia	2 (6.1)	8 (24.2)	10 (30.3)
Cough	9 (27.3)	0 (0.0)	9 (27.3)
Diarrhea	9 (27.3)	0 (0.0)	9 (27.3)
Neutropenia	0 (0.0)	9 (27.3)	9 (27.3)
Thrombocytopenia	0 (0.0)	9 (27.3)	9 (27.3)
Vomiting	9 (27.3)	0 (0.0)	9 (27.3)
Anemia	0 (0.0)	8 (24.2)	8 (24.2)
Nausea	8 (24.2)	0 (0.0)	8 (24.2)
Abdominal pain	7 (21.2)	0 (0.0)	7 (21.2)
Back pain	6 (18.2)	1 (3.0)	7 (21.2)
Chills	7 (21.2)	0 (0.0)	7 (21.2)
Decreased appetite	7 (21.2)	0 (0.0)	7 (21.2)
Hypokalemia	5 (15.2)	2 (6.1)	7 (21.2)
Pneumonia	4 (12.1)	2 (6.1)	6 (18.2)
Most frequently reported drug-related AEs,^¶^ n (%)			
Fatigue	8 (24.2)	1 (3.0)	9 (27.3)
Neutropenia	0 (0.0)	9 (27.3)	9 (27.3)
Nausea	8 (24.2)	0 (0.0)	8 (24.2)
Thrombocytopenia	0 (0.0)	4 (12.1)	4 (12.1)
Neutropenic sepsis^#^	0 (0.0)	1 (3.0)	1 (3.0)

† Adverse events were coded with MedDRA, version 16.1.

‡ All Grades include Grade 1–4 events only; Grade 5 events include neutropenic sepsis (possibly drug-related; further details below) and pneumonia (due to progressive disease).

§ Adverse events include those preferred terms reported for at least seven (21.2%) subjects in the study (N = 33). Subjects with more than one AE within a MedDRA system organ class and preferred term were counted once for the worst grade within that system organ class and preferred term.

¶ Adverse events include those preferred terms reported for at least four (12.1%) subjects and the single subject with a drug-related death (Grade 5). Subjects with more than one AE within a MedDRA system organ class and preferred term were counted once for the worst grade within that system organ class and preferred term.

# There was one Grade 5 drug-related event (1/33 patients, 3.0%; not including deaths related to disease progression), which included neutropenic sepsis reported in a patient with AML who received the highest dose of 2 mg talazoparib; this was considered to be possibly drug-related by the investigator. Further details on the patient's medical history can be found in the Supplementary Material.

AE: Adverse event; AML: Acute myeloid leukemia; MedDRA: The Medical Dictionary for Regulatory Activities; TEAE: Treatment-emergent adverse event.

Of the Grade 3/4 TEAEs reported for 28 (84.8%) patients, the most frequently reported were neutropenia, thrombocytopenia, febrile neutropenia, and anemia (Table 3-4). Drug-related Grade 3/4 TEAEs were reported for 14 (42.4%) patients (Table 3). The most frequently reported drug-related Grade 3/4 TEAEs were neutropenia and thrombocytopenia (Table 4).

Serious AEs (SAEs) were reported for 25 (75.8%) patients, of which 17 (51.5%) patients had a Grade 3/4 SAE and 3 (9.1%) patients had a drug-related SAE (two patients reported neutropenic sepsis [grade 3 and 5, respectively], one patient reported shortness of breath [grade 2] and neutropenic fever [grade 4]; Table 3). SAEs in the infections and infestations system organ class were the most common in both cohorts (including neutropenic sepsis, pneumonia, respiratory tract infection, and bacteremia). At least one TEAE leading to dose interruption and/or reduction was observed for 15 patients (45.5%). For ten patients (30.3%), TEAEs leading to dose interruption/reduction were reported to be related to study drug, and were primarily hematologic in nature (Table 3).

### Deaths

Thirteen (39.4%) deaths were reported (Table 3; three patients with AML and one with MDS died during the study; eight patients with AML and one with MCL died of disease progression within 30 days after the last study drug dose). All deaths were either related to the advanced nature of underlying hematologic malignancies impairing hematologic and immunologic functions (10/13 patients), or to consequent infection, febrile neutropenia, or neutropenic sepsis (3/13 patients). One death due to neutropenic sepsis, which occurred in a patient with AML who received the highest dose of 2 mg talazoparib, was considered to be possibly drug-related by the investigator. This patient had a history of neutropenia and thrombocytopenia and, at study baseline, had Grade 4 leukopenia, neutropenia, and thrombocytopenia; further details on the patient's medical history can be found in the Supplementary Material.

### PK analysis

Following administration of multiple once-daily doses of talazoparib (cycle 2 day 1), PK analysis showed rapid absorption across the 100–2000 μg dose range and linear PK (Table 5; Supplementary Figure 1). Changes in PK and pharmacodynamics parameters were not specifically analyzed among the relapsed, refractory, and newly diagnosed condition. Results for urinary elimination of the parent compound suggest urinary elimination is the major clearance pathway for talazoparib (Table 5).

**Table 5.** Pharmacokinetic parameters in plasma and urine after multiple daily talazoparib dosing.

	Parameter summary statistics^†^ by dose, mg/day						
	0.10	0.20	0.30	0.45	0.90	1.35	2.00
Plasma PK	n = 6^‡^	n = 3	n = 4	n = 2	n = 4	n = 3	n = 2
T_max_, hr	1.53 (0.250, 2.02)	0.983 (0.550, 1.03)	1.63 (0.917, 2.98)	4.50 (1.00, 8.00)	1.48 (1.00, 2.00)	1.03 (1.00, 4.00)	1.00 (1.00, 1.00)
C_max_, ng/ml	1.280 (30.9)	3.130 (25.8)	3.460 (49.9)	8.090 (59.7)	12.20 (52.2)	19.70 (33.0)	30.50 (114)
AUC_0–24_, ng•hr/ml	16.80 (31.2)	48.60 (21.6)	45.50 (32.6)	111.0 (26.6)	142.0 (63.3)	226.0 (16.0)	454.0 (128)
AUC_0–t_, ng•hr/ml	16.70 (26.7)	49.60 (20.7)	36.50 (26.6)	111.0 (26.6)	138.0 (65.1)	225.0 (15.9)	405.0 (126)
AR	7.87 (5.55, 8.46)	6.79 (5.76, 7.19)	8.57 (4.56, 14.1)	8.03 (7.57, 8.48)	5.15 (1.92, 16.7)	3.80 (3.28, 4.22)	3.62 (0.490, 6.75)
Urine PK	n = 4^§^	n = 2	n = 2	n = 2	n = 5^¶^	n = 2	n = 2
Ae_0–24_, mg	0.043 (56.4)	0.090 (35.3)	0.162 (30.1)	0.408 (33.7)	0.369 (49.5)	0.531 (29.8)	0.533 (116)
Fe_0–24_	0.428 (56.4)	0.450 (35.3)	0.539 (30.1)	0.906 (33.7)	0.410 (49.5)	0.393 (29.8)	0.267 (116)
ARC_0–24_, l/hr	3.51 (57.2)	1.78 (52.4)	4.96 (59.9)	3.64 (7.47)	3.56 (45.8)	2.44 (9.09)	1.66 (45.5)

† Geometric mean (geometric CV%) for all parameters except median (range) for T_max_ and AR.

‡ n = 4 for AUC_0–24_ and AR.

§ n = 2 for ARC_0–24_.

¶ n = 4 for ARC_0–24_.

Ae_0–24_: Amount of drug excreted into the urine within the dose interval, 0 to 24 h; AR: Accumulation ratio; ARC_0–24_: Average renal clearance from time 0 to 24 h post-dose; AUC_0–24_: Area under the curve from time 0 to 24 h; AUC_0–t_: Area under the curve from 0 to last quantifiable sampling point post-dose; C_max_: Maximum concentration; CV: Coefficient of variation; Fe_0–24_: Fraction of administered drug excreted from time 0 to 24 h; PK: Pharmacokinetics; T_max_: Time to maximum plasma concentration.

### Anti leukemic activity

Stable disease was reported for 18 (54.5%) patients. Stable disease of at least 16 weeks' duration was reported in six patients (cohort 1, two patients with AML and three with MDS; cohort 2, one patient with MCL; Table 6). Transfusion independence was reported for two patients, including one with MDS and one with AML.

**Table 6.** Best overall response with stable disease following talazoparib.

	Cohort 1			Cohort 2		
	AML (n = 21)	MDS (n = 4)	All (n = 25)	CLL (n = 4)	MCL (n = 4)	All (n = 8)
Stable disease, n (%)	9 (42.9)	4 (100)	13 (52.0)	3 (75.0)	2 (50.0)	5 (62.5)
Stable disease ≥16 weeks,^†^ n (%)	2 (9.5)^‡^	3 (75.0)^§^	5 (20.0)	0	1 (25.0)	1 (12.5)

† Duration of stable disease is defined for a subject whose best overall response is stable disease as the time from the date of the first dose to the date of the first documentation of progressive disease or treatment failure. If a subject did not have a date of any documentation of progressive disease or treatment failure, the duration of stable disease is defined as the time from the date of the first dose to the date of the last tumor assessment as a censored value.

‡ One patient became transfusion independent after five treatment cycles.

§ One patient became transfusion independent after two treatment cycles.

AML: Acute myeloid leukemia; CLL: Chronic lymphocytic leukemia; MCL: Mantle cell lymphoma; MDS: Myelodysplastic syndrome.

## Discussion

Despite the fact that several pre-clinical studies have demonstrated promising results for PARP inhibitors in hematologic malignancies, there remains an insufficiency of data on the clinical potential of PARP inhibition in these diseases. This Phase I study in hematologic malignancies, conducted between July 2011 and November 2013, aimed to evaluate the MTD of talazoparib monotherapy, as well as provide preliminary results on safety, PK, and antileukemic activity.

Based on the relatively limited, but important, data presented in this study, the MTD in patients with hematologic malignancies appears to be consistent with an MTD of 1 mg once daily reported previously in solid tumors [22]. In cohort 1 of this study, the MTD was exceeded at 2 mg once daily due to dose-limiting febrile neutropenia and fatal neutropenic sepsis, both reported in one patient. The MTD was determined as 1.35 mg once daily because no DLTs were observed in the three patients treated with this dose level. While the MTD was not definitively elucidated in cohort 2 of this study, it was exceeded at 0.9 mg once daily due to dose-limiting Grade 4 neutropenia, which was reported for two patients. As the MTD of talazoparib monotherapy is well established in solid tumors at 1 mg/day in a continuous dosing schedule [22], doses above this, such as those investigated in this study, are likely to have limited clinical applications.

The AEs and DLTs observed in this study were consistent with a study population comprised of patients with advanced hematologic malignancies and potential disease-related hematologic and immunologic dysfunction. The most common Grade 3 and higher AEs were primarily hematologic events (anemia, neutropenia, and thrombocytopenia) or their immediate consequences (e.g., febrile neutropenia). Similarly, the four DLTs reported were also primarily, or related to, hematologic toxicity (neutropenia, febrile neutropenia, and neutropenic sepsis). Overall, the AE profile observed in this study is generally consistent with that reported in solid tumors [22].

Talazoparib demonstrated favorable PK properties with rapid absorption and dose-proportional increases in total exposure over a wide dose range (0.1–2.0 mg/day) after multiple daily doses. These PK results are consistent with those reported previously in solid tumors [22,35].

While single-agent talazoparib demonstrated modest anti leukemic activity in this small, heavily pre-treated, molecularly unselected group of patients, there were promising signals of stable disease and transfusion independence. Indeed, stable disease was reported in over half (18/33) of patients, including two patients with transfusion independence. Disease stability lasting at least 16 weeks was reported in six patients. Overall, these observations suggest that further research of PARP inhibitors in hematologic malignancies may be warranted, particularly to investigate rational combination therapies and identify molecular signatures that may identify patients likely to respond to treatment.

Although molecular signatures are of interest to potentially identify patients likely to benefit from PARP inhibition, there are currently limited clinical data from patients with hematologic malignancies. In a Phase I dose-escalation study in CLL or MCL patients, a trend towards improved overall survival was observed with olaparib in patients with *ATM* mutations versus those without *ATM* mutations; however, this was not statistically significant [36]. It is possible that results from ongoing clinical trials will provide further insight into appropriate patient selection. For example, ongoing trials are assessing the effect of talazoparib monotherapy in cohesin-mutated AML or MDS with excess blasts (NCT03974217), as well as olaparib monotherapy in *IDH*-mutated AML or MDS (ClinicalTrials.gov Identifier: NCT03953898).

As hematologic malignancies represent a heterogeneous group of cancers with complex etiology [1], the combination of PARP inhibitors with other therapies, such as DNA demethylating agents, may increase therapeutic efficacy and overcome potential resistance mechanisms [37]. In AML, talazoparib is currently under investigation in combination with several therapies, including decitabine in untreated, relapsed, or refractory AML (ClinicalTrials.gov Identifier: NCT02878785), as well as with antibody-drug conjugate gemtuzumab ozogamicin in CD33-positive relapsed or refractory AML (NCT04207190). PARP inhibitors enhance response to immunotherapy by promoting neoantigen release, increasing tumor mutational burden and increasing the expression of immune checkpoint regulators such as PD-L1 and CTLA-4 [38]; while promising results have been reported in solid tumors [38], further research in hematologic malignancies may also be relevant.

The results presented here are limited by the variety of molecularly unselected hematologic malignancies, as appropriate for the dose-finding primary objective of this Phase I study, and the genetic signatures of the patient's leukemias are unknown. Additionally, the majority (90.9%) of the patients in the study were white; as there are known racial/ethnic disparities in the incidence and clinical outcomes for patients with hematologic malignancies [39], future studies would benefit by a more inclusive study population. With a clinically diverse population and small patient numbers, however, it is difficult to determine anti leukemic activity. Nevertheless, these limited data suggest that with appropriate patient selection, a clearer signal could be identified.

## Conclusion

In conclusion, the AEs and DLTs reported in this study were consistent with the underlying nature of the population's disease. The MTD in patients with hematologic malignancies appears to be in a similar range (∼1 mg monotherapy, once daily) to that previously reported in the parallel Phase I study of talazoparib in solid tumors [22]. While limited, single-agent talazoparib in this small, heavily pretreated, molecularly unselected group showed some promising anti leukemic signals of stable disease and transfusion independence. Therefore, future studies involving PARP inhibitors and patients with hematologic malignancies should continue to investigate rational combination therapies and identify patients likely to benefit from treatment.

## Future perspective

Hematologic malignancies are genetically heterogeneous and prone to accumulate DNA damage, providing a therapeutic opportunity for PARP inhibition [40–44]. The use of PARP inhibitors combined with established agents to overcome resistance mechanisms is promising [43]. leukemias including AML, chronic myelogenous leukemia and myeloproliferative disorder have complex mutational landscapes that can evolve over time, and PARP inhibition is an attractive means of exploiting defects in cells that may carry DNA damage response and homologous recombination repair deficiencies due to chromosomal instability [45–48]. PARP inhibitors are currently under investigation for the treatment of these leukemias in combination with decitabine, temozolomide, carboplatin, and topotecan [48]. Combinations with epigenetic modifiers such as histone deacetylase inhibitors may soon follow [48,49].

Future clinical studies are needed to determine if PARP inhibitor combination therapies will be effective in treating patients with hematologic malignancies similar to observations made in solid tumors [5,6]. Clinicians will need to weigh the potential benefits of PARP inhibitors for patients with hematologic malignancies against the potential risks – a recent study showed that patients receiving PARP inhibitors had an increased risk of developing MDS and AML [50]. However, continued study of PARP inhibitors is warranted in patients with hematologic malignancies, particularly as part of a rational combination therapy that may lead to better disease control, especially in patients with poor-risk cytogenetics [48].

**Summary points**

- This Phase I, two-cohort, dose-escalation trial (ClinicalTrials.gov Identifier: NCT01399840) evaluated talazoparib monotherapy in molecularly unselected patients with advanced hematologic malignancies.
- The primary objective of the study was to establish the maximum tolerated dose (MTD) of talazoparib in patients with acute myeloid leukemia or myelodysplastic syndrome (cohort 1; n = 25) and in patients with chronic lymphocytic leukemia or mantle cell lymphoma (cohort 2; n = 8).
- The secondary objectives were to assess the safety, pharmacokinetics, and preliminary anti-leukemic activity of talazoparib in both cohorts.
- Results demonstrated that the MTD was exceeded at 2.0 mg/day in cohort 1 and at 0.9 mg/day in cohort 2. Grade ≥3 adverse events were primarily hematologic.
- Stable disease was reported for 18/33 (54.5%) patients, and transfusion independence was achieved in two patients.
- Based on the data presented here, the MTD of talazoparib in patients with hematologic malignancies appears to be consistent with the MTD of 1 mg once daily reported in solid tumors.
- Talazoparib was relatively well tolerated and the AEs observed were consistent with the underlying nature of the population's disease.
- Despite the small, heavily pretreated, molecularly unselected group of patients in this study, talazoparib demonstrated anti leukemic activity leading to stable disease and transfusion independence.
- Overall, these results suggest that further clinical trials and translational research of poly(ADP-ribose) polymerase inhibitors in hematologic malignancies are warranted to ascertain the efficacy of this class of drug in hematologic malignancies.
