# Supplementary figures and images for: A Phase I trial of talazoparib in patients with advanced hematologic malignancies
Source: Int J Hematol Oncol. 2021 Oct 22;10(3):IJH35. doi: 10.2217/ijh-2021-0004 (PMC8609999; doi:10.2217/ijh-2021-0004)

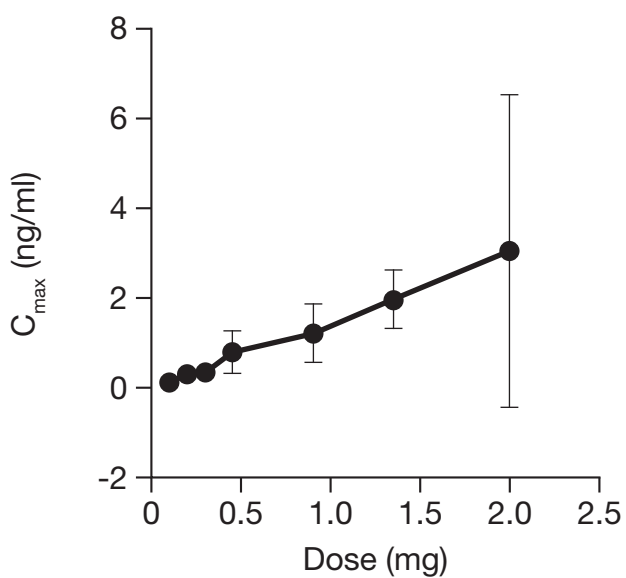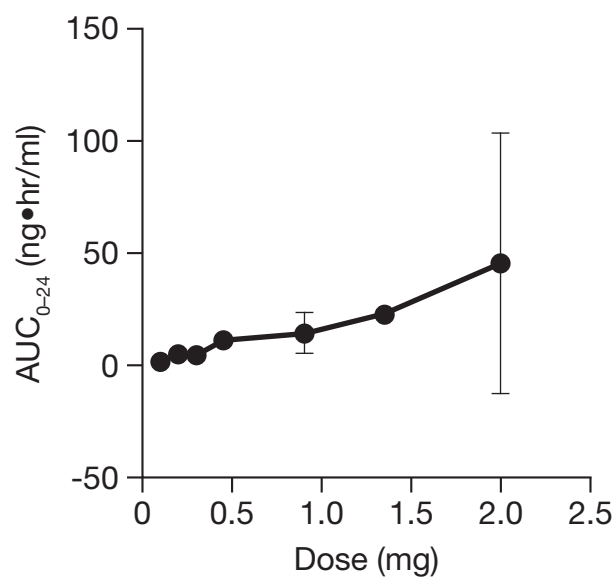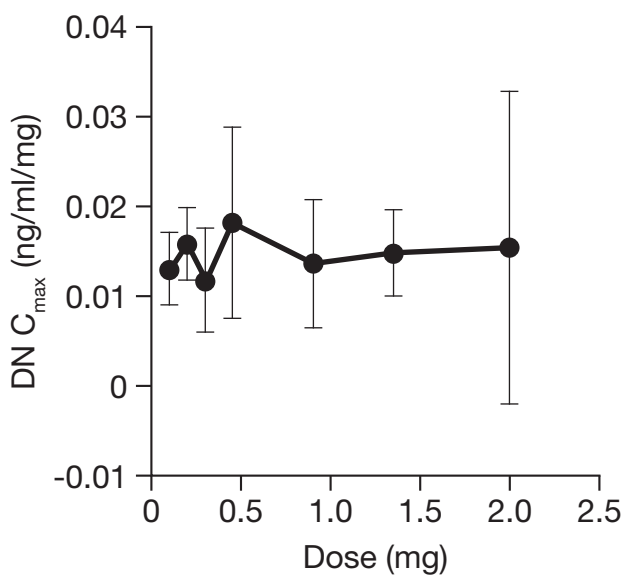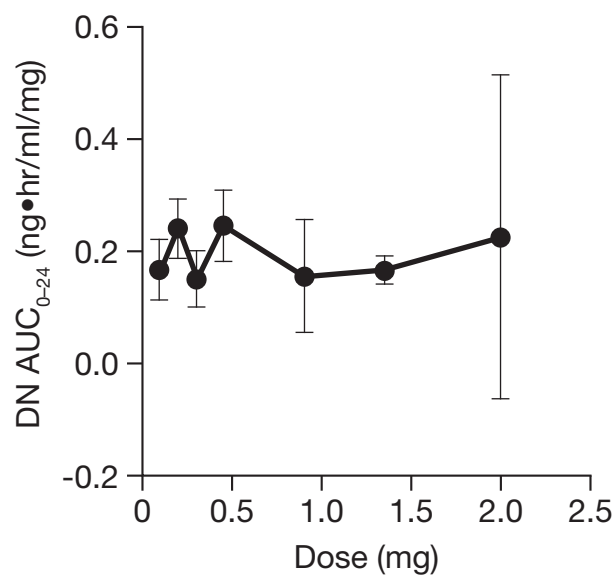

Supplement: Supplementary file 1 [file ijh-10-35-s1.pdf]
