# Supplementary material for: A Phase I trial of talazoparib in patients with advanced hematologic malignancies
Source: Int J Hematol Oncol. 2021 Oct 22;10(3):IJH35. doi: 10.2217/ijh-2021-0004 (PMC8609999; doi:10.2217/ijh-2021-0004)
Supplement: Supplementary file 2 [file ijh-10-35-s2.docx]

**Supplementary material**

**Medical history of the patient in Cohort 1 who experienced possible drug-related Grade 5 neutropenic sepsis**

One case of neutropenic sepsis, which was eventually fatal, occurred in a 71-year-old male in Cohort 1 who was receiving the highest dose of 2 mg talazoparib once daily.

The patient was diagnosed with *de novo* AML in 2010, and had relapsed AML at study entry. His last chemotherapy regimen was in June 2012, which included clofarabine, cytarabine, and filgrastim. He had a medical history of neutropenia and thrombocytopenia and, at study baseline, he had had Grade 4 leukopenia, neutropenia, and thrombocytopenia.

The patient received 2 mg talazoparib once daily between 17 January 2013 and 5 February 2013. On 4 February, he was admitted with neutropenic sepsis, and on 6 February, he was diagnosed with possible pneumocystis carinii pneumonia (PCP). The patient died on 12 February 2013 (7 days after the last dose of study drug). Cause of death was reported as PCP but without radiological or microbiological confirmation. The investigator assessed the neutropenic sepsis as possibly related to talazoparib treatment. Other etiological factors included the underlying AML.

**Supplementary figure legends**

**Supplementary Figure 1.** Dose linearity and proportionality in plasma following multiple doses of talazoparib.

Talazoparib plasma C_max_ (top left), DN C_max_ (bottom left), AUC_0–24_ (top right), and DN AUC_0–24_ (bottom right) following daily doses ranging from 0.1 to 2 mg/day. Filled circles represent the mean value at each dose level and error bars represent the standard deviations.

AUC_0–24_: area under the curve from time 0 to 24 hours; C_max_: maximum concentration; DN: dose normalized.
