# Supplementary material for: A Phase I trial of talazoparib in patients with advanced hematologic malignancies
Source: Int J Hematol Oncol. 2021 Oct 22;10(3):IJH35. doi: 10.2217/ijh-2021-0004 (PMC8609999; doi:10.2217/ijh-2021-0004)
Supplement: Supplementary file 3 [file ijh-10-35-s3.docx]

**Supplementary Table 1.** Patient disposition by dose level and diagnosis (safety population).^†^

|  | **Cohort 1** | | | **Cohort 2** | | | **Overall   (N = 33)** |
| --- | --- | --- | --- | --- | --- | --- | --- |
| **Dose level, mg/day** | **AML (n = 21)** | **MDS (n = 4)** | **All (n = 25)** | **CLL (n = 4)** | **MCL (n = 4)** | **All (n = 8)** |  |
| 0.10 | 3 (14.3) | 0 (0.0) | 3 (12.0) | 1 (25.0) | 2 (50.0) | 3 (37.5) | 6 (18.2) |
| 0.20 | 2 (9.5) | 1 (25.0) | 3 (12.0) | 0 (0.0) | 0 (0.0) | 0 (0.0) | 3 (9.1) |
| 0.30 | 3 (14.3) | 2 (50.0) | 5 (20.0) | 0 (0.0) | 0 (0.0) | 0 (0.0) | 5 (15.2) |
| 0.45 | 3 (14.3) | 0 (0.0) | 3 (12.0) | 0 (0.0) | 0 (0.0) | 0 (0.0) | 3 (9.1) |
| 0.90 | 4 (19.0) | 0 (0.0) | 4 (16.0) | 3 (75.0) | 2 (50.0) | 5 (62.5) | 9 (27.3) |
| 1.35 | 3 (14.3) | 0 (0.0) | 3 (12.0) | 0 (0.0) | 0 (0.0) | 0 (0.0) | 3 (9.1) |
| 2.00 | 3 (14.3) | 1 (25.0) | 4 (16.0) | 0 (0.0) | 0 (0.0) | 0 (0.0) | 4 (12.1) |

^†^Data presented as n (%).

AML: acute myeloid leukemia; CLL: chronic lymphocytic leukemia; MCL: mantle cell lymphoma;
MDS: myelodysplastic syndrome.
