# Supplementary material for: A Phase I trial of talazoparib in patients with advanced hematologic malignancies
Source: Int J Hematol Oncol. 2021 Oct 22;10(3):IJH35. doi: 10.2217/ijh-2021-0004 (PMC8609999; doi:10.2217/ijh-2021-0004)
Supplement: Supplementary file 4 [file ijh-10-35-s4.docx]

**Supplementary Table 2.** Exposure to talazoparib (safety population).

|  | **Cohort 1** | | | | | | | | **Cohort 2** | | |
| --- | --- | --- | --- | --- | --- | --- | --- | --- | --- | --- | --- |
| **Dose level, mg/day** | **0.10**  **(n = 3)** | **0.20**  **(n = 3)** | **0.30**  **(n = 5)** | **0.45**  **(n = 3)** | **0.90**  **(n = 4)** | **1.35**  **(n = 3)** | **2.00**  **(n = 4)** | **All**  **(n = 25)** | **0.10**  **(n = 3)** | **0.90**  **(n = 5)** | **All**  **(n = 8)** |
| **Total duration of study drug exposure,^†^ days** | | | | | | | | |  |  |  |
| Mean (SD) | 112.0 (71.0) | 191.3 (253.7) | 161.8 (156.5) | 28.0 (14.0) | 32.5 (18.3) | 76.0 (33.2) | 88.8 (131.5) | 100.6 (124.6) | 32.3  (3.8) | 62.0 (79.2) | 50.9 (61.9) |
| Median (range) | 72.0 (70.0, 194.0) | 56 .0 (34.0, 484.0) | 91.0 (49.01, 434.0) | 28.0 (14.0, 42.0) | 37.5  (7.0, 48.0) | 66.0 (49.0, 113.0) | 24.5 (20.0, 286.0) | 49.0  (7.0, 484.0) | 34.0 (28.0, 35.0) | 36.0 (13.0, 202.0) | 34.5 (13.0, 202.0) |
| **Total amount of doses received, mg** | | | | | | | | |  |  |  |
| Mean (SD) | 11.0 (7.3) | 59.6 (87.9) | 57.7 (73.7) | 16.1 (11.2) | 26.9 (14.7) | 102.6 (44.8) | 67.3 (43.1) | 49.3 (53.2) | 3.1  (0.55) | 28.3 (21.6) | 18.9 (20.9) |
| Median (range) | 7.2 (6.5, 19.4) | 11.0  (6.8, 161.1) | 27.3 (9.9, 187.8) | 13.7 (6.3, 28.4) | 31.3 (6.3, 38.7) | 89.1 (66.2, 152.6) | 49.0 (40.0, 131.0) | 28.4 (6.3, 187.8) | 3.4 (2.5, 3.5) | 19.1  (10.8, 64.1) | 13.1  (2.5, 64.1) |
| **Mean daily dose, mg/day** | | | | | | | | |  |  |  |
| Mean (SD) | 0.10 (0.00) | 0.25 (0.08) | 0.33 (0.07) | 0.54 (0.12) | 0.89 (0.02) | 1.35 (0.00) | 1.68 (0.65) | 0.74 (0.61) | 0.10 (0.00) | 0.77 (0.24) | 0.52 (0.39) |
| Median (range) | 0.10 (0.10, 0.10) | 0.20 (0.20, 0.33) | 0.30 (0.30, 0.46) | 0.49 (0.45, 0.68) | 0.90 (0.86, 0.90) | 1.35 (1.35, 1.35) | 2.00 (0.70, 2.00) | 0.49 (0.10, 2.00) | 0.10 (0.10, 0.10) | 0.90 (0.34, 0.90) | 0.57 (0.10, 0.90) |
| **Total number of initiated treatment cycles** | | | | | | | | |  |  |  |
| Mean (SD) | 6.0 (3.5) | 9.7 (12.4) | 7.4 (6.7) | 1.7 (0.6) | 1.8 (0.5) | 4.3 (1.5) | 3.3 (3.9) | 4.9 (5.6) | 2.0 (0.0) | 3.2 (3.8) | 2.8 (3.0) |
| Median (range) | 4.0  (4.0, 10.0) | 3.0  (2.0, 24.0) | 5.0  (2.0, 19.0) | 2.0  (1.0, 2.0) | 2.0  (1.0, 2.0) | 4.0  (3.0, 6.0) | 1.5  (1.0, 9.0) | 3.0  (1.0, 24.0) | 2.0  (2.0, 2.0) | 2.0  (1.0, 10.0) | 2.0  (1.0, 10.0) |
| **Total number of completed treatment cycles** | | | | | | | | |  |  |  |
| Mean (SD) | 5.0 (3.5) | 8.7 (12.4) | 7.2 (6.8) | 1.0 (1.0) | 1.3 (1.0) | 3.3 (1.5) | 3.0 (4.0) | 4.3 (5.6) | 1.0 (0.0) | 2.4 (3.8) | 1.9 (2.9) |
| Median (range) | 3.0  (3.0, 9.0) | 2.0  (1.0, 23.0) | 4.0  (2.0, 19.0) | 1.0  (0.0, 2.0) | 1.5  (0.0, 2.0) | 3.0  (2.0, 5.0) | 1.0  (1.0, 9.0) | 2.0  (0.0, 23.0) | 1.0  (1.0, 1.0) | 1.0  (0.0, 9.0) | 1.0  (0.0, 9.0) |

^†^Total duration of study drug exposure = last dose date – first dose date + 1.

SD: standard deviation.
